# Biocompatibility and antimicrobial effect of demineralised dentin matrix hydrogel for dental pulp preservation

**DOI:** 10.1007/s10266-024-00994-2

**Published:** 2024-09-14

**Authors:** Nessma Sultan, Josette Camilleri, Ben A. Scheven

**Affiliations:** 1 Department of Oral Biology, Faculty of Dentistry, Mansoura University, Mansoura, Egypt; 2 Oral Biology and Dental Morphology, Faculty of Dentistry, Mansoura National University, Gamasa, 7731168 Egypt; 3 School of Dentistry, College of Medical and Dental Sciences, University of Birmingham, Birmingham, UK; 4 School of Dentistry, Oral Biology, College of Medical and Dental Sciences, University of Birmingham, Birmingham, UK

**Keywords:** Dentin, Dental pulp stem cells, Demineralised dentin matrix hydrogel, Hydrogels, Mineral trioxide aggregate, Pulp capping agents, Regenerative endodontics

## Abstract

Regeneration of dentin and preserving pulp vitality are essential targets for vital pulp therapy. Our study aimed to evaluate a novel biomimetic pulp capping agent with increased dentin regenerative activities. To produce demineralised dentin matrix (DDM) particles, human extracted teeth were ground and treated with ethylene diamine tetra-acetic acid solution. DDM particles were added to sodium alginate and this combination was dripped into a 5% calcium chloride to obtain DDM hydrogel (DDMH). The eluants of both DDMH and mineral trioxide aggregate (MTA) were tested using an MTT assay to detect their cytotoxic effect on dental pulp stem cells (DPSC). Collagen-I (COL-I) gene expression was analysed on DPSC exposed to different dilutions of pulp capping material eluants by real-time quantitative polymerase chain reaction. Acridine orange staining was used to monitor the cell growth over the tested materials. Agar diffusion assay was utilised to test the antibacterial effect of DDMH and MTA compared to controls. MTT assay revealed that neat eluates of DDMH promoted DPSC viability. However, neat eluates of MTA were cytotoxic on DPSC after 72 h of culture. Moreover, DPSC were capable of growth and attached to the surface of DDMH, while they showed a marked reduction in their number when cultured on the MTA surface for one week, as shown by the acridine orange stain. In DPSC cultured with DDMH eluates, the COL-I gene was overexpressed compared to those cultured with MTA eluants. DDMH had significant antimicrobial activity in comparison to MTA after 24 h incubation. This in vitro study showed that DDMH could be an alternative pulp capping agent for regenerative endodontics.

## Introduction

Dentin and dental pulp are two specialised tissues usually recognised as the dentin-pulp complex. According to the hydrodynamic theory of hypersensitivity, sensitive dentin is permeable throughout the length of dentinal tubules. This permits bacterial products’ diffusion across the dentin to the dental pulp and thus can irritate pulpal tissues. One of the most significant issues in dentistry is maintaining the dentin-pulp complex’s integrity and vitality [1–3].

Dental pulp inflammation occurs due to bacterial invasion before and after cavity preparation. To eliminate severe dental pulp infection, dentists may eventually resort to removing the pulp (i.e., pulpectomy). If the latter is not carried out, ischaemia can develop due to impaired perfusion and the resulting pulp necrosis and periapical disease [4]. Following pulpectomy, root canal therapy (RCT) is performed and the pulp chamber and roots are filled with inorganic materials (paste and gutta percha). The RCT-treated tooth is devitalised and brittle, with a high risk of post-operative fracture. Therefore, an effective therapeutic approach is essential for preserving pulp vitality. Advances in tissue engineering and regenerative medicine have provided possibilities for regenerative endodontics [5]. In regenerative endodontics, the main objectives are the replacement of inflamed/necrotic pulp tissue with regenerated pulp-like tissue, pulp–dentin complex and also the replacement of damaged coronal dentin to revitalise teeth [6].

In dental practice, direct pulp capping (DPC) is performed to prevent pulp death. Materials used for vital pulp therapy that are directly applied over dental pulp and localised under the permanent restoration should have nontoxic, antibacterial, anti-inflammatory, and good sealing properties and ideally have the ability to induce reparative dentinogenesis and dentin mineralisation [7].

Calcium hydroxide, Ca(OH)_2_, is no longer recommended for vital pulp therapy, as indicated by the European Society of Endodontology guidelines and also the American Association of Endodontists [8, 9]. Ca(OH)_2_ induces coagulative necrosis and pathological calcifications, leading to the obliteration of the pulp chamber [10–13]. The materials indicated for vital pulp therapy are hydraulic calcium silicate cements with MTA, which exhibit an enhanced reparative capacity for dentin without pulp inflammation. Nonetheless, it is difficult to handle this material and poorly adheres to the tooth [14–17]. Irrespective of which material is applied and unlike the natural dentin-pulp complex formation, the reparative capacity of dentin involves the pulp reaction to chemical stimulation with a high pH material [18–21]. Thus, attempts are made to find a novel DPC material with minimal adverse effects that promote pulpal repair.

Natural proteins and bioactive molecules in the dentin matrix have broad application prospects in dentin regeneration [22, 23]. Many studies have been conducted to produce dentin matrix-based formulations with optimal mechanical and handling characteristics. Adding some excipients, e.g., alginate-based hydrogel, can provide the material with good handling characteristics [24, 25]. In the DPC technique, scaffolds for dentin regeneration have been made from various materials. These components may promote mineralisation and cell development, but they may not be able to induce differentiation towards an odontogenic specialisation [26, 27]. The finest option for creating the ideal dentin regeneration mimic is a dentin tissue scaffold. Demineralised dentin matrix hydrogel (DDMH), an innovative injectable mixture that combines partial DDM particles and alginate hydrogel as the matrix phase, has been shown as a potential material for the restoration of bone and dentin defects [28–30]. Almost all studies regarding DDMH concerned with the partially demineralised dentin particles [28–31], while no available studies on completely demineralised dentin particles have been reported so far. The capacity to modify the degree of demineralisation of dentin particles opens up new avenues for developing more robust osteoinductive materials. Thus, our study aimed first to adjust the level of dentin particle demineralisation with preservation of protein content, then tested the biocompatibility of the DDMH on DPSC in addition to assessing their antimicrobial effect to be used for vital pulp therapy inducing dentin regeneration.

## Materials and methods

Ten (*n* = 10) permanent first premolars with no caries were obtained from either male or female patients aged 11–18 years old from the outpatient clinic of the Faculty of Dentistry, Mansoura University, Egypt. These teeth underwent extraction for orthodontic reasons. The collection and storage of teeth were performed in accordance with infection control standards after obtaining approval from the Faculty of Dentistry ethical committee with number A24060722. We aimed for a significance level of 5% and a confidence interval of 95%. From prior experience, the data were obtained from three independent experiments with three replicates for each group/experiment.

### Isolation and characterisation of DPSC

DPSC have been isolated according to previously published articles [32, 33]. Extracted teeth were disinfected and then rinsed with phosphate buffer saline (PBS). Under copious water supply, a cut was performed around the cementoenamel junction utilising a sterilised dental bur (MANI, Inc. US) with a high-speed handpiece (NSK, US) to expose the pulp chamber. Teeth were put into transport media containing basic medium Dulbecco modified essential medium/ F12 with l-glutamine (DMEM/F12) (Life Science, UK) supplemented with 20% foetal bovine serum (FBS) (Life Science, UK) along with penicillin and streptomycin (100 µg/mL each) (Biowest, US). All processing procedures were aseptically undertaken using a 100 mm petri plate (Sterilin) in a biohazard laminar flow hood.

Dental pulp tissue was removed via endodontic H-file #30 (MANI, Inc, US). The isolated pulp tissue was minced and plated in a T-25 flask (Greiner Bio-one, Germany) containing the above-mentioned media. Culture was done at 37 °C in an incubator with 5% CO_2_. Cultures were assessed daily under an inverted microscope (Olympus Corp, US) to detect contamination and cell outgrowth.

Cultures were rinsed twice with PBS, trypsinised with 0.05% trypsin–EDTA (Biowest, USA) for 2–5 min, and neutralised with 10% FBS containing DMEM/F12. Detached cells were collected in a tube and underwent centrifugation at 226×*g* for 5 min. Cells underwent resuspension either in a growth medium or kept in a freezing medium containing 90% FBS and 10% dimethyl sulfoxide (DMSO) (Sigma Aldrich) in liquid nitrogen (− 196 °C) until analysis.

Cells grown till they reached 70% confluency were trypsinised and resuspended in a growth medium. Equal volumes of cell suspension and 0.4% trypan blue (Gibco, Fisher Scientific, Scotland, UK) were mixed. A haemocytometer was used to count viable and non-viable cells.

### Characterisation

To examine surface molecular expression in DPSC, 5 × 10^5^ third-passage cells were fixed with 4% paraformaldehyde for 15 min. Following washing with PBS, they were labelled with primary CD105, CD73, CD90, CD34 and CD45 antibodies for 1 h, then labelled with fluorescein isothiocyanate-conjugated secondary antibodies in the dark for 45 min. The cells that stained positive for CD90, CD105, and CD73, and those stained negative for CD34 and CD45, were evaluated with FACS calibre flow cytometry (BD Immunocytometry Systems).

### Dentin particle preparation (Fig. 1)

**Fig. 1:**
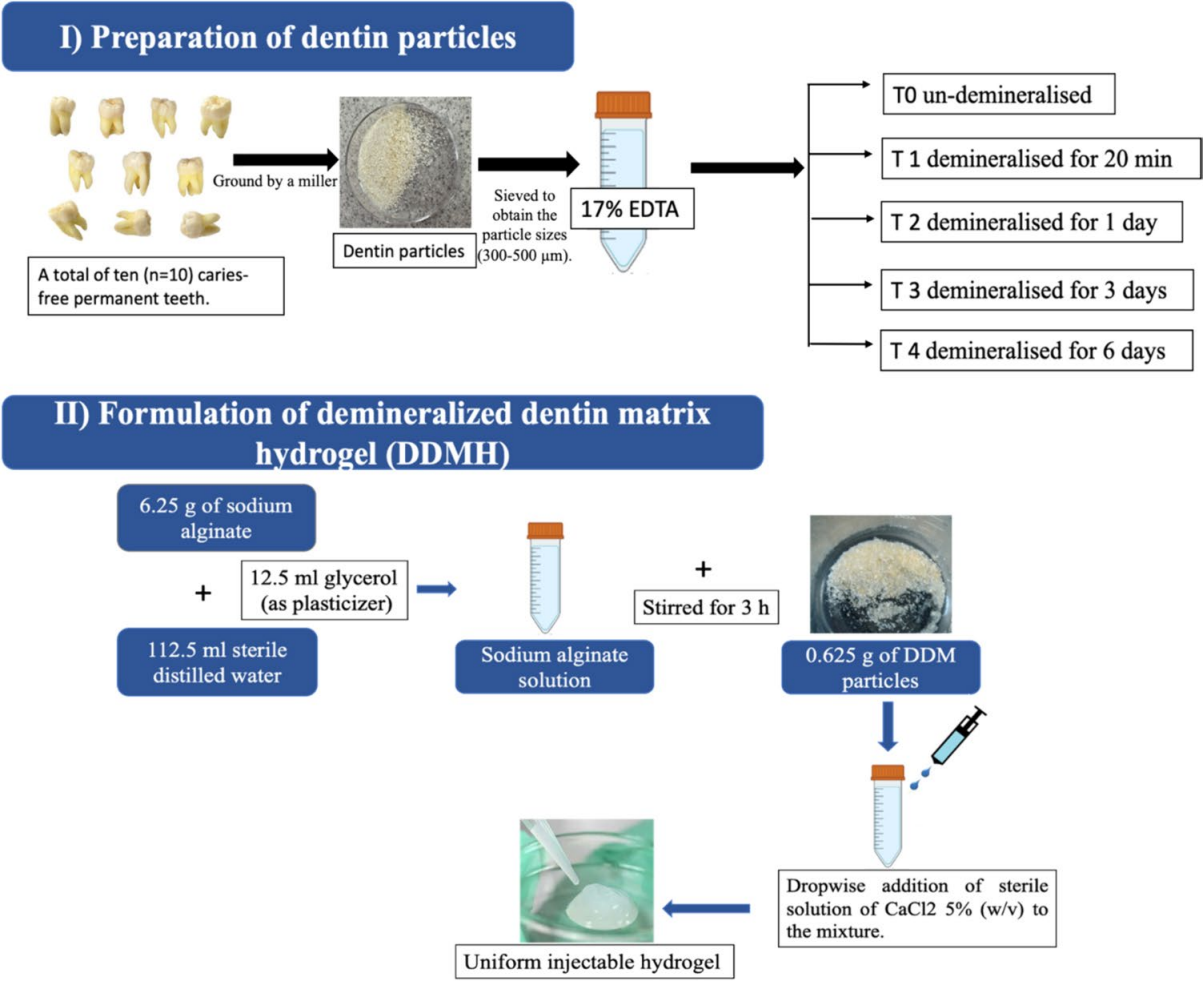
Schematic structure showing step by step formulation of DDMH

Dentin particles were prepared as described previously [30, 34]. After tooth extraction, a dental drill was used to remove enamel and root cementum. Then, teeth were transferred to a sterile container and ground by a miller into particles and sieved using a sieve system (Millburn, New Jersey, USA) to obtain particle sizes of 300 µm–500 µm.

### Dentin particle demineralisation (Fig. 1)

The milled dentine particles were treated with 17% EDTA at pH 7.0 and used at different application times to optimise the preservation of the bioactive organic component. The dentin particles were dried and randomly divided into the following 5 groups (10 g each): T0 (control or un-demineralised); T1 (demineralised for 20 min); T2 (demineralised for 1 day); T3 (demineralised for 3 days); T4 (demineralised for 6 days). After the incubation, EDTA was discarded and specimens were washed twice in PBS, after which the particles were dried and sterilised with ultraviolet (UV) radiation for 15 min and underwent storage at 4 °C.

### SEM and energy dispersive X-ray analysis (SEM–EDS)

To evaluate the morphological surface characteristics and chemical composition of dentin particles, SEM (Jeol JSM 6510, Jeol, Peabody, MA) was utilised and connected to an electron detector for EDS (Oxford X-Max 20 INCA, UK). After gold-sputtering, dentin particles were mounted onto SEM stubs and examined using an accelerating voltage of 15 kV.

### Analysis of COL-I, BMP-2, and VEGF content in DDM particles

ELISA was performed for quantification of collagen-I (COL-I), bone morphogenetic protein-2 (BMP-2) and vascular endothelial growth factor (VEGF) content in demineralised and control dentin samples. Briefly, ≈50 mg of dentin particles were transferred into a 1.5 mL polypropylene tube, and 500 μL of extraction buffer (50 mM HEPES, pH 7.4; 1 mM phenylmethylsulfonyl fluoride; 2 μg/mL leupeptin; 2 μg/mL aprotinin; 1 μg/mL pepstatin and 1% [*v*/*v*] Triton X-100). Incubation was done at 4 °C overnight, and after 3 freezing/thawing cycles, samples were centrifuged, and the supernatant was collected as a protein extract [35]. For the ELISA assay, 15 μg of the protein extract was added, and the tests were performed in accordance with the manufacturer’s guidelines (Human COL1A1 (Collagen Type I Alpha 1) ELISA Kit Cat No.EH0958, Fine Biotech, Wuhan, Hubei, China & Human VEGF ELISA Kit, Cat No.EH0327, Fine Biotech, Wuhan, Hubei, China & Human BMP-2 ELISA Kit, cat No. E-EL-H0011, Elab-science Biotechnology, USA.). Results from the ELISA tests were normalised to the weight of each sample.

### Formulation of human demineralised dentin matrix hydrogel (Fig. 1)

Under sterile conditions, 6.25 g of sodium alginate was dispersed in 112.5 ml distilled water and 12.5 ml glycerol and mixed overnight to obtain a 5% (*w*/*v*) sodium alginate solution. Then, 0.625 g of DDM particles was dispersed in this solution with a mass ratio of 1:10, the mixture was stirred for 3 h, and then 10 cycles of centrifugation were done at 1506×*g* for 5 min. After that, the hydrogel was obtained by dropwise addition of a sterile solution of CaCl_2_ 5% (*w*/*v*) to the mixture. At the end of the process, hydrogel centrifugation was performed for 2 cycles at 1506×*g* for 5 min to obtain uniform injectable hydrogel mass [34].

### Pulp capping material disc eluants

Elution studies were conducted to test the cytotoxicity of the DDMH and the MTA pulp capping material. MTA (MTA, Angelus, Londrina, Brazil) was mixed, following the manufacturers’ guidelines. 100 μl of freshly mixed MTA and DDMH were added in 96-well-plates, sterilised using UV for 15 min, and kept in RT for 48 h to achieve a complete setting, then 200 µl of serum-free DMEM/F12 was added to the sterile discs of either MTA or DDMH placed in 96 well-plates and underwent incubation at 37 °C in a humid atmosphere with 5% CO2. After 24 h, the disc eluants/extracts of the tested materials were collected under a sterile condition and filtered in sterile filters (diameter = 0.22 μm) [36]. The total protein content in the collected eluants/extracts was measured using bicinchoninic acid assay (BCA) kits (Thermo Scientific, Pierce BCA, Illinois, USA). The data were obtained from 3 independent experiments with 3 replicates for each group/experiment.

### MTT assay

The cytotoxic effects of a series of concentrations of tested material eluants were tested by the MTT assay (MTT Cell Growth Kit; Chemicon, Rosemont, IL). In brief, DPSC were seeded at a density of 4000 cells/cm^2^ on a 96-well plate in 200 μL DMEM/F12 supplemented with 10% FBS. Complete culture medium was replaced 24 h post-seeding by 200 μL of a series of concentrations of tested material eluants (25%, 50%, 75% and 100%) and underwent incubation for 72 h. MTT was added at a concentration equal to 1 mg/mL for 4 h. Then, the culture medium containing MTT was removed, followed by adding 100 μL DMSO to release formazan. A microplate reader (ELx800; Bio-Tek Instruments, Winooski, VT) was utilised to measure the absorbance at 570 nm using 690 nm absorbance as the reference wavelength. DPSC cultured in a serum-free medium were utilised as a control group.

#### RT-qPCR

Cells cultured in a T-75 flask in a series of concentrations of tested material eluants were homogenised. Total RNA underwent extraction using Direct-zol RNA miniprep plus (Cat# R2072, Zymo Research Corp., US). Then, quantity and quality were determined using a dual spectrophotometer (Beckman US, Indianapolis, Indiana, US). Super Script IV One-Step RT-PCR kit (Catalogue# 12,594,100, Thermo Fisher, Waltham, MA US) was utilised for RT-qPCR. 96-well plate step one instrument (Applied Biosystem, US) was used in a thermal profile under the following conditions: 10 min at 45 ºC for reverse transcription, 2 min at 98 ºC for RT inactivation and initial denaturation by 40 cycles (each = 10 s) at 98 ºC, 10 s at 55 ºC and 30 s at 72 ºC for amplification. After the run, data were described in Cycle threshold (Ct) for target genes and the housekeeping gene. Normalisation for variation in the expression of each target gene; COL-I was carried out referring to the mean critical threshold (CT) expression value of the GAPDH housekeeping gene by the ΔΔCt method. The relative quantitation of each target gene was determined through the calculation of the 2-∆∆Ct method. The sequence of the primer was forward 5′-GTACATCAGCCCAAACCCCAAG-3′ and reverse 5′-CGGAACCTTCGCTTCCATACTC-3′ (Gene bank accession number: XM_032912698.1) for COL-I gene. GAPDH was used as a housekeeping gene (Gene bank accession number: XM_017592435.1). The data were obtained from 3 independent experiments with 3 replicates for each group/experiment.

#### Acridine orange (AO) staining

Freshly mixed MTA and DDMH were added to 96-well-plates, sterilised using UV for 15 min, and kept in RT for 48 h to achieve a complete setting. DPSC were seeded at a concentration of 4000 cells/cm^2^ in a 96-well plate over the sterile discs of MTA and DDMH and were incubated in 200 µl of complete culture media at 37 °C in a humid atmosphere with 5% CO_2_. Cell numbers were counted after 3 and 7 days of culture time by AO (Sigma-Aldrich Cat. No 318337). Briefly, an aliquot of 1 µl of 0.5 mg/ml AO reagent was added to wells and underwent incubation for 10 min at RT. Green fluorescence was evaluated with a fluorescent microscope with an excitation wavelength of 460 nm. The experiment was performed in triplicates, and cells were counted per microscopic field.

#### Evaluation of antimicrobial effects of pulp capping material discs

The microorganisms used in our study include gram-positive bacteria Streptococcus mutans and Staphylococcus aureus. These microorganisms were grown in tryptic–soy broth and adjusted to the turbidity of a 0.5 McFarland BaSo_4_ standard (1.5 × 108 CFU⁄ml). Five Petri dishes were utilised for 24 h evaluation. Seeding was done utilising sterile cotton-tipped applicators brushed on the agar’s surface. Four wells were punched in each agar plate and filled with pulp capping materials in addition to two types of antibiotics, IPM and vancomycin, as positive controls. The plates were then maintained in an incubator at 37 °C in a humid atmosphere. 24 h later, the antimicrobial effect of either DDMH or MTA was detected by measuring the diameter of inhibition zones on plates.

#### Statistical analysis

The Shapiro–Wilk test was used to test the data for normality. One-way analysis of variance (ANOVA) with Tukey’s multiple comparison post-test was utilised to test for the significance between groups (*P* < 0.05) in MTT, RT-qPCR and AO tests. While the Kruskal–Wallis Test was utilised, Mann–Whitney post hoc analysis adjustment was carried out to determine the significance among groups (*P* < 0.05) in ELISA analysis and agar diffusion assay. Data were analysed using the SPSS 20.0.0 statistical software (IBM, Chicago, US).

## Results

### Isolation and characterisation of DPSC

**Fig. 2:**
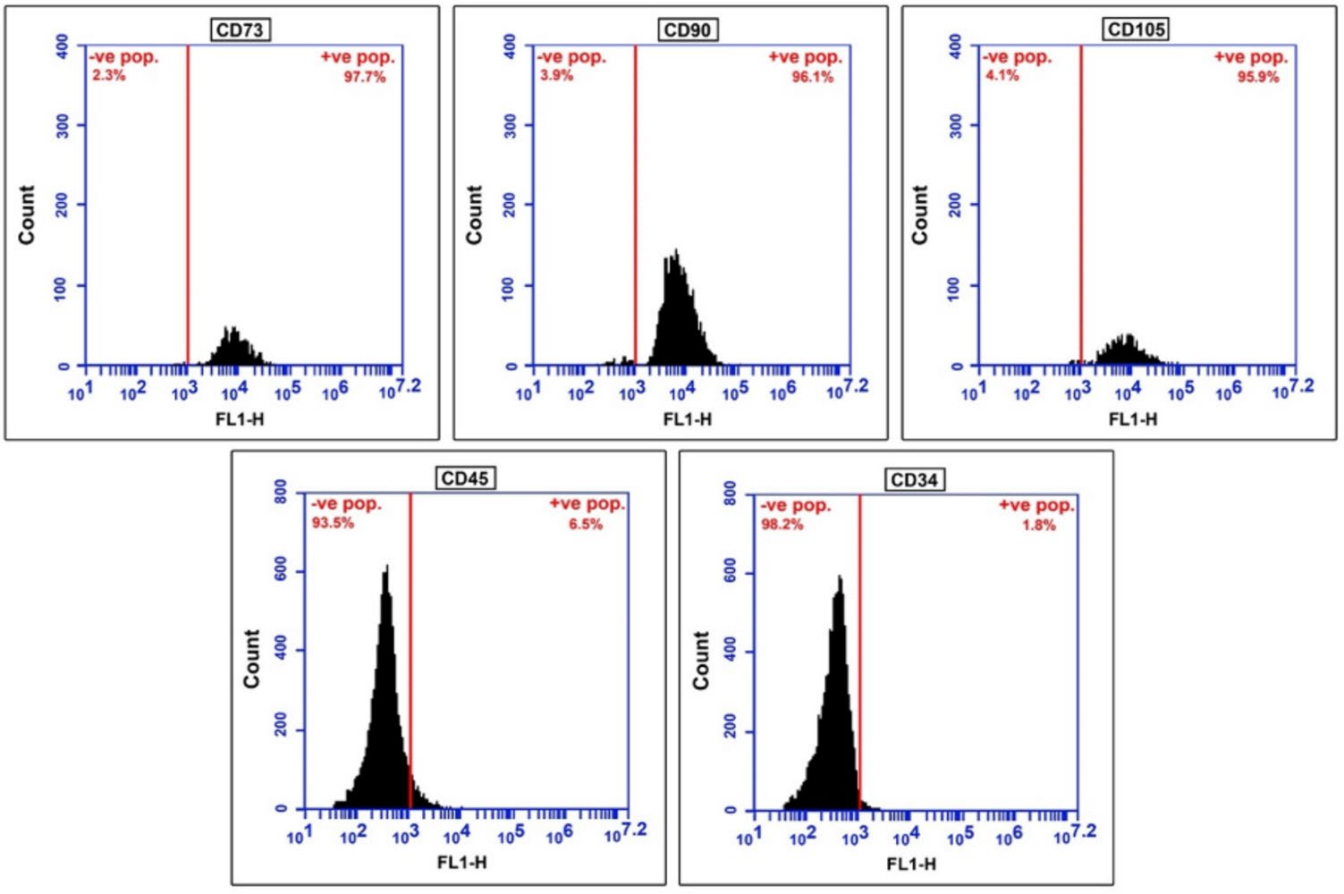
Mesenchymal phenotype analysis of DPSC by flow cytometry. Histograms display fluorescence intensity as measurement parameter on the x-axis and the cell count on the y-axis

DPSC displayed a spindle-shaped morphology. Cells in the third passage were collected for phenotypic characterisation using flow cytometry (Fig. 2). Our data confirmed that > 95% of the cells expressed the mesenchymal stem cell markers CD73, CD90, and CD105 whilst being negative for the haematopoietic markers CD34 and CD45.

### Effect of demineralisation on morphological surface properties and chemical composition of dentin particles

**Fig. 3:**
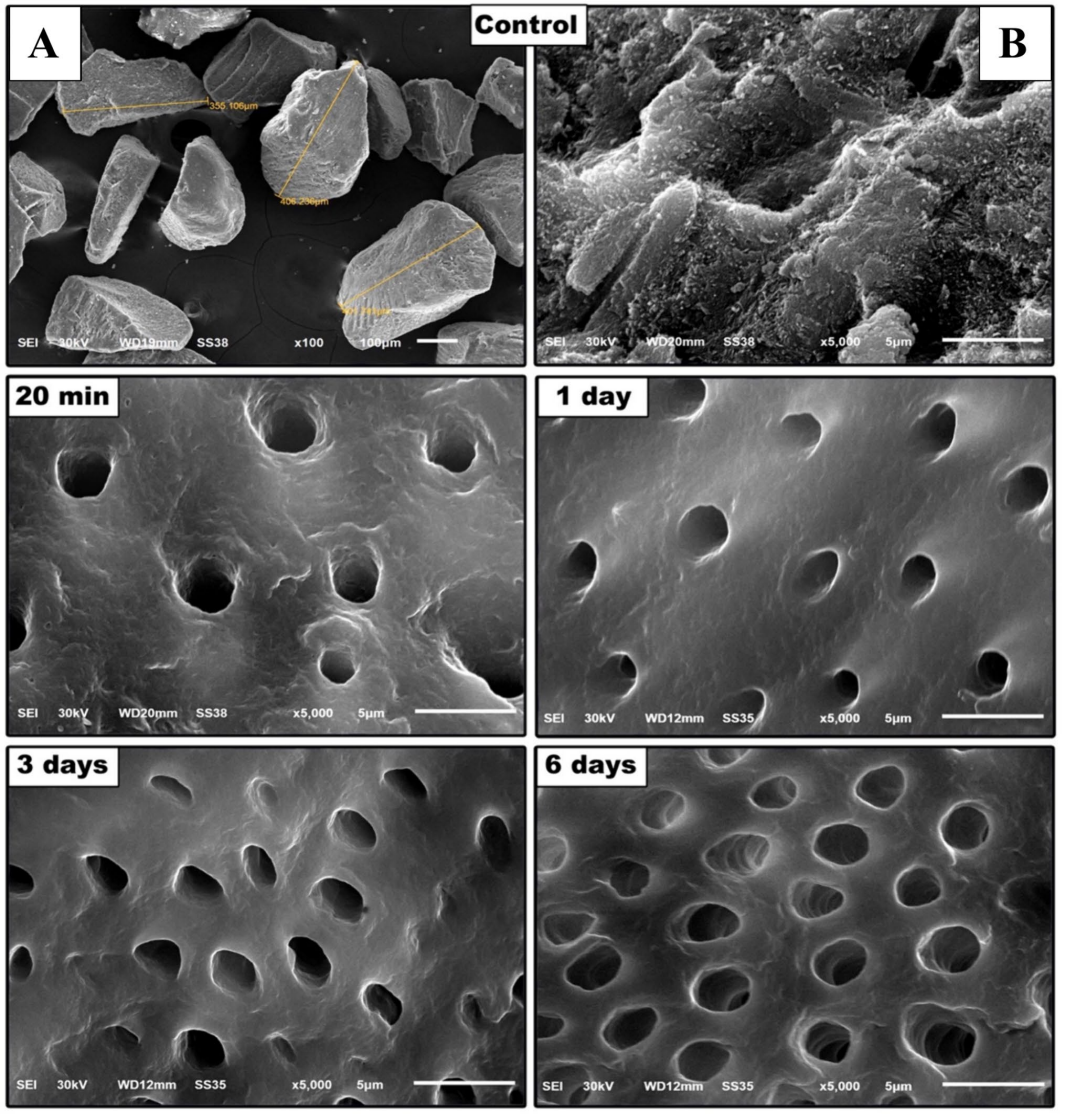
SEM images of dentin particles after different demineralisation times. In the control group (without demineralisation); A; shows the particle size, which was ranging from 300 to 500 μm. B; shows occlusion of dentinal tubules with smear layer. After demineralisation for 20 min and 1 day, the dentinal tubules were exposed, while after 3 and 6 days of demineralisation, the number of exposed dentinal tubules obviously increased

SEM analysis demonstrated that the size of dentin particles was in the range of 300–500 μm (Fig. 3). SEM micrographs demonstrated mineral crystals in the control group surrounding the rim of dentinal tubules (Fig. 3); this was confirmed by the Ca and P peaks in the EDS spectrum in the control dentin samples (Table 1). Demineralised dentin samples showed a significant removal of mineral components, giving a highly smooth dentin surface (Fig. 3). EDS analysis revealed significant alterations in Ca and P content following demineralisation (Table 1). The reduction in % Ca and % P was more than 50% in demineralised dentin samples compared to their control counterparts.

**Table 1:** EDS of the demineralised dentin surfaces

Sample/content	C %	O %	P %	Ca %
Control	13.31 ± 0.21	41.30 ± 0.38	14.70 ± 0.24	25.89 ± 0.75
20 min	55.28 ± 2.08	27.11 ± 0.34	2.13 ± 0.21	4.38 ± 0.14
1 day	54.93 ± 2.10	26.35 ± 0.48	1.19 ± 0.16	3.47 ± 0.16
3 days	61.63 ± 0.48	27.74 ± 0.10	0.30 ± 0.35	1.98 ± 0.48
6 days	62.49 ± 2.70	29.47 ± 1.20	7.44 ± 0.29	0.61 ± 0.28

Within the window of analysis, 4 random locations were measured. An elemental analysis spectrum was obtained for all locations. Data are expressed as means and SD

### Effect of demineralisation on COL-I, VEGF, and BMP-2 contents in dentin particles

COL-I, VEGF and BMP-2 content were quantified in dentin particles. ELISA analysis revealed higher levels of COL-I, VEGF, and BMP-2 in demineralised samples than in non-treated particles; probably, the detection of proteins becomes easier following the demineralisation process. Of note, great amount of COL-I was detected in samples demineralised for 3 days than in the other treated samples, but the difference was not significant. This suggests that demineralisation process did not cause damage to the ECM of dentin. A similar trend was also observed between demineralised and un-demineralised particles regarding BMP-2 and VEGF content (Table 2). Significantly higher BMP-2 levels were detected in samples demineralised for 3 and 6 days compared with samples demineralised only for either 20 min or 1 day, while significantly higher VEGF levels were observed in dentin samples demineralised for 3 days compared with other treated and control samples.

**Table 2:** Protein level of COL-1, BMP-2 and VEGF detected in demineralised and control dentin samples as determined by ELISA

Groups (mean rank)	n		Control	20 min	1 day	3 days	6 days	P value^a^
COL-1	3	*P* value^b^	3.84	6.65 P1 = 0.008	6.86 P2 = 0.008 P5 = 0.08	7.72 P3 = 0.008 P6 = 0.007 P8 = 0.008	6.51 P4 = 0.008 P7 = 0.07 P9 = 0.08 P10 = 0.007	< 0.001***
BMP-2	3	*P* value^b^	178	302 P1 = 0.008	340 P2 = 0.008 P5 = 0.08	542 P3 = 0.008 P6 = 0.007 P8 = 0.008	538 P4 = 0.008 P7 = 0.007 P9 = 0.008 P10 = 0.09	< 0.001***
VEGF	3	*P* value^b^	20.08	56.75 P1 = 0.008	75.92 P2 = 0.008 P5 = 0.08	119.25 P3 = 0.008 P6 = 0.008 P8 = 0.008	12.58 P4 = 0.08 P7 = 0.05 P9 = 0.05 P10 = 0.008	< 0.001***

Kruskal–Wallis, Mann–Whitney test. P^1^: comparison between control vs. 20 min; P^2^: comparison between control vs. 1 day; P^3^: comparison between control vs. 3 days; P^4^: comparison between control vs. 6 days; P^5^: comparison between 20 min vs. 1 day; P^6^: comparison between 20 min vs. 3 days, P^7^: comparison between 20 min vs. 6 days, P^8^: comparison between 1 day vs. 3 days, P^9^: comparison between 1 day vs. 6 days, P^10^: comparison between 3 days vs. 6 days, P: probability

Significance levels were set at *P < 0.05, **P < 0.01, ***P < 0.001

^a^Kruskal–Wallis

^b^Mann–Whitney

Based on DDM characterisation results, demineralisation of dentin particles using EDTA for 3 days resulted in a minimal amount of minerals (*P*: 0.30%, Ca: 1.98%) and sufficient amounts of proteins (COL-I: 7.72 ng/dl, BMP-2: 542 pg/dl, VEGF: 119.25 pg/dl). Thus, DDM after 3 days of demineralisation was used in all the following experiments, and the other demineralisation times were omitted.

### Cytotoxicity of pulp capping material disc eluants

**Fig. 4:**
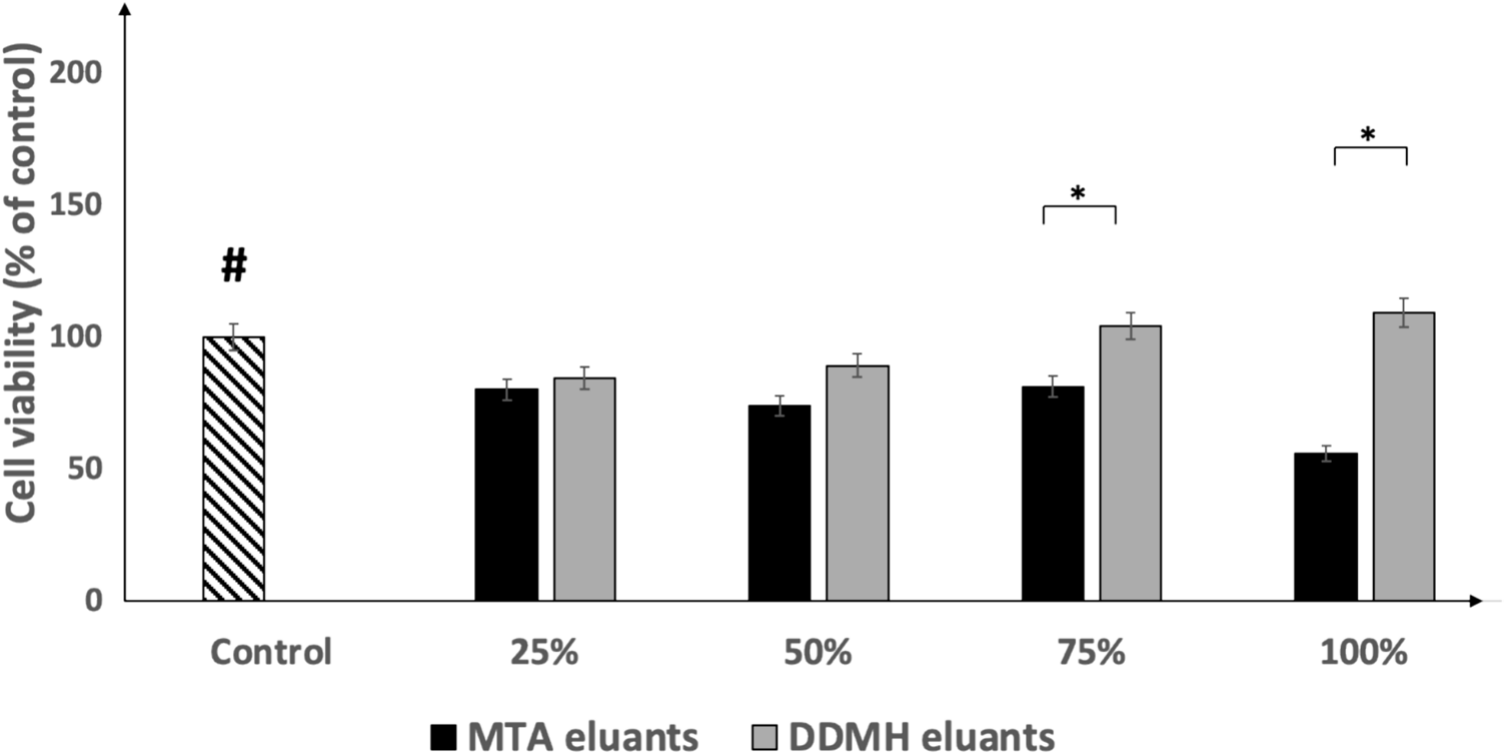
MTT analysis of viable DPSC number cultured with either DDMH or MTA eluants. DPSC were exposed to serum-free medium (control), 25%, 50%, 75% and 100% DDMH or MTA disc eluants for 72 h. Data are presented as mean ± SD; *n* = 3. * *P* < 0.05. **#** Means significant to 100% MTA eluants* P* < 0.05

The protein concentration of the tested materials eluants was analysed by BCA. The total protein content in DDMH eluants was 1000 µg/ml, while only a trace amount of protein could be detected in MTA eluants. Then, an MTT assay was used to test the metabolic activity of DPSC seeded with various concentrations of tested material eluants for 72 h (Fig. 4). Preliminary work indicated that short incubation period did not lead to cell death, so the length of the incubation was extended to 72 h. Compounds with little cytotoxic action on cells need longer incubation durations.

MTT assay demonstrated a significant difference in cell viability among study groups exposed to neat concentration and 75% eluants of the DDMH and MTA (*P* < 0.05). However, at concentrations of 50% and 25%, the proliferation of cells did not significantly differ between tested materials (*P* > 0.05). The percentage of viability of cells exposed to DDMH eluants at neat concentration was 109%, which showed a 9% increase compared to the control group seeded in serum-free medium; however, such an increase was non-significant.

The neat concentration of MTA eluants was cytotoxic to DPSC after 72 h of incubation, being significantly different from the serum-free control group. However, this material, in concentrations of 25%, 50%, and 75%, showed a reduction in cell viable number in comparison with control samples, which was statistically non-significant (*P* > 0.05).

### Impact of pulp capping material disc eluants on COL-I gene expression in DPSC

**Fig. 5:**
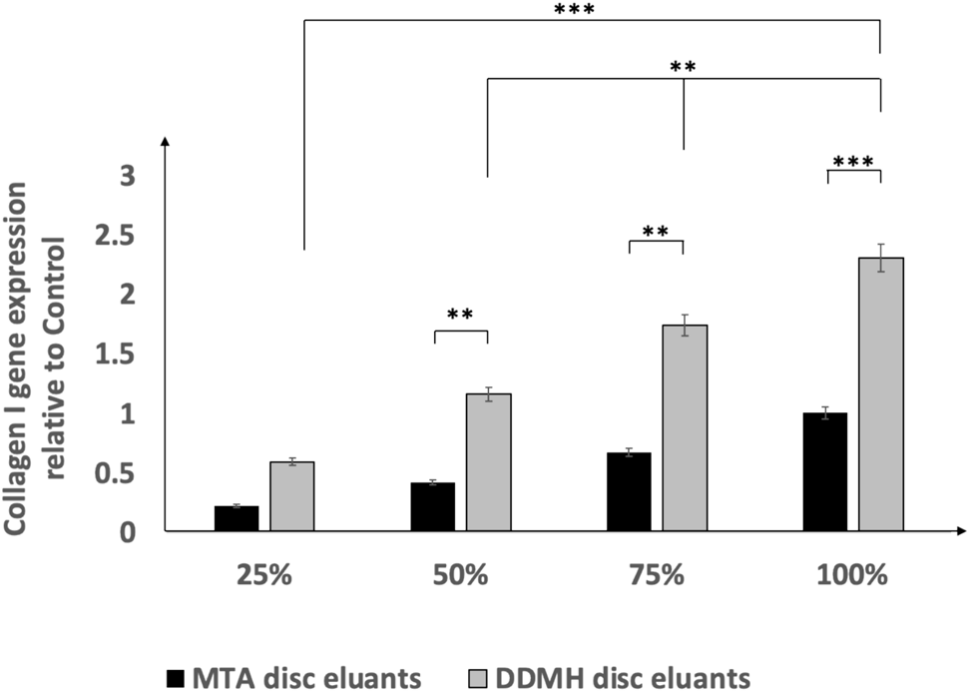
RT-qPCR analysis of COL-1 gene expression in DPSC incubated with either 25%, 50%, 75% and 100% of DDMH or MTA eluants for 3 weeks. Data are presented as mean ± SD relative to control. Housekeeping gene is GAPDH; *n* = 3. **P < 0.01, *** < *P* 0.001

RT-qPCR was performed to quantify the expression of the COL-I gene in DPSC incubated with various concentrations of tested material disc eluants for 21 days. DPSC seeded in 100% DDMH eluants not only showed significantly higher expression of COL-I in comparison to all other dilutions but also showed significantly higher expression than cells seeded with MTA eluants. Our findings revealed that the COL-I gene demonstrated a significant overexpression in DPSC cultured with DDMH eluants in comparison with those cultured in the eluants of MTA (Fig. 5).

### Effect of pulp capping material discs on DPSC numbers

**Fig. 6:**
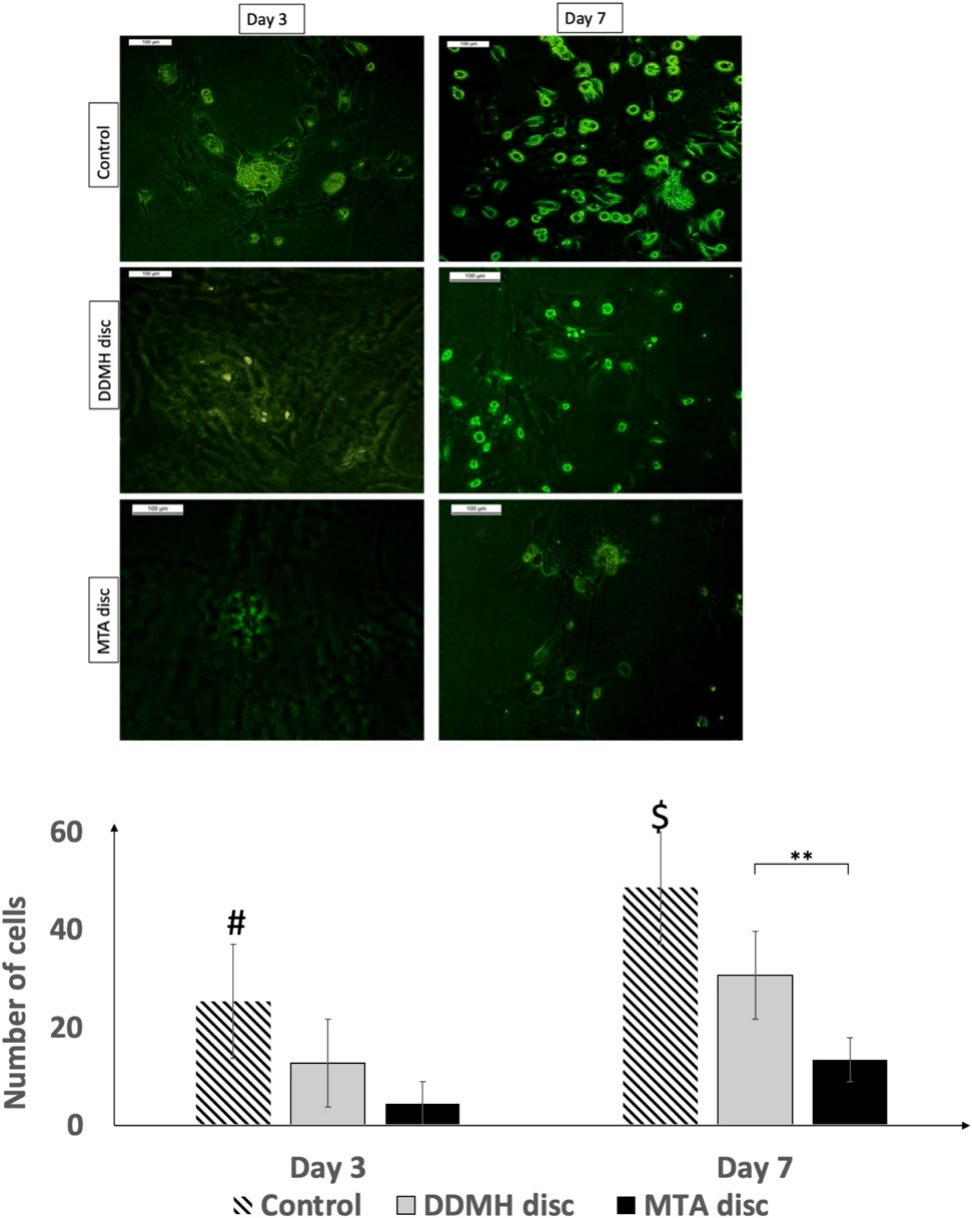
Assessment of DPSC growth and proliferation on tested material discs using acridine orange, fluorescent staining. DPSC were seeded on either DDMH, or MTA disc and cell attachment was assessed over 3 and 7 days of culture. Control group represents cells seeded in well plates with complete culture medium. Data are presented as mean ± SD of cells/field; n = 3. # Means significant in comparison to DDMH and MTA discs at day 3. $ means significant in comparison to DDMH and MTA discs at day 7. ** < *P* 0.01

The growth of DPSC on the tested material discs was evaluated after 3 and 7 days of culture using AO fluorescent staining. Our results revealed that the number of attached cells on the two tested material discs increased over time. The control specimens demonstrated the highest mean number of cells after culture for 1 week, but the number of cells seeded on the DDMH disc was significantly higher than what was seen with MTA discs (Fig. 6).

### Antimicrobial effects of pulp capping material discs

**Fig. 7:**
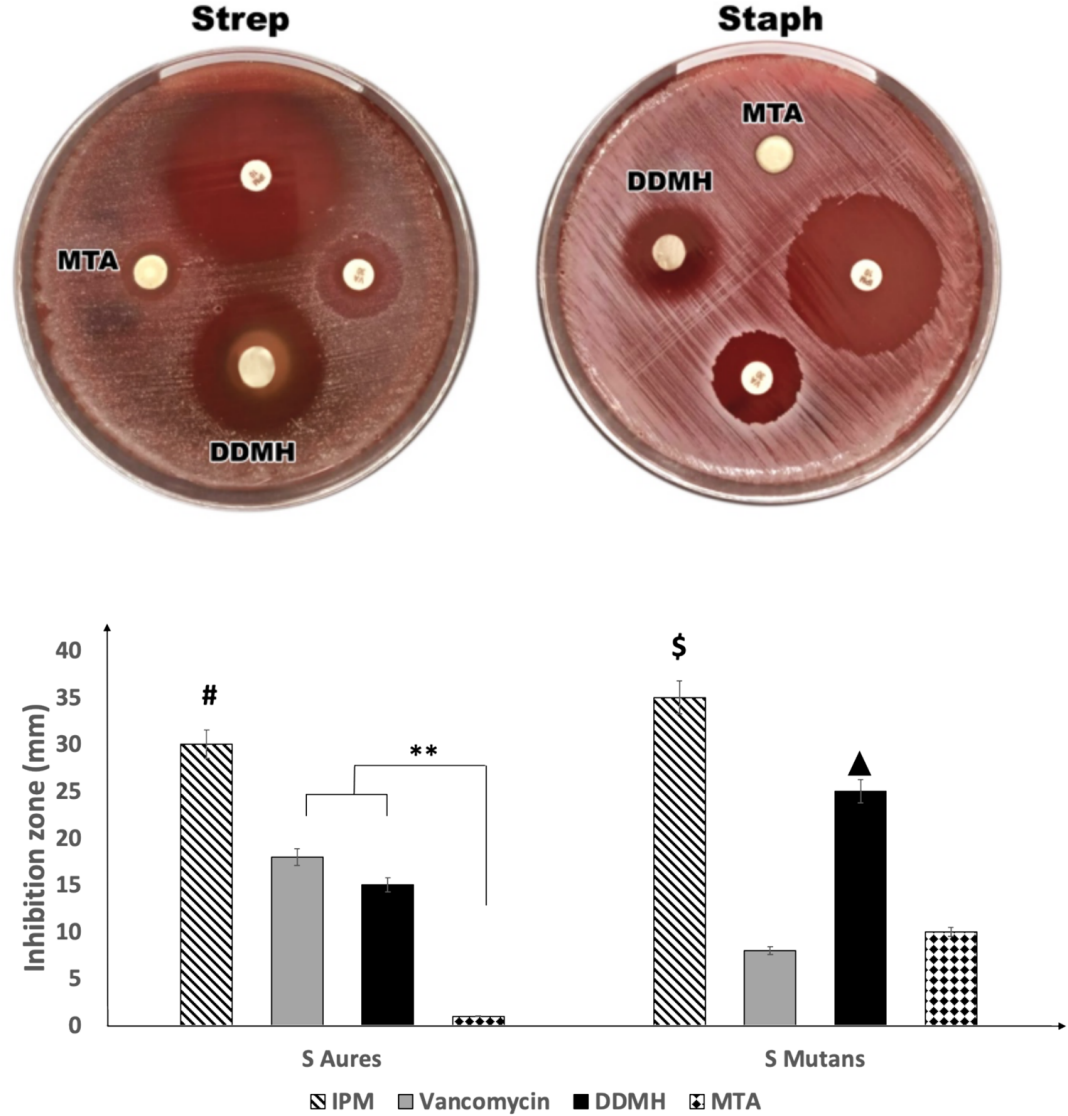
Agar diffusion test to measure the diameter of inhibition zone of MTA and DDMH on bacterial growth; *streptococcus mutans* and *staphylococcus aureus*. The bar chart shows the mean ± SD; *n* = 3 of the inhibition zone diameter around the tested materials. IPM and vancomycin antibiotics were used as controls. # & means significant to vancomycin, DDMH and MTA, while $ means significant to vancomycin and MTA. means significant to vancomycin and MTA. ** < P 0.01

To study the effects of DDMH on microbial growth, an agar diffusion assay was performed with IPM and vancomycin as a positive control. Table 3 and Fig. 7 show the antibacterial activity of the tested materials. The MTA disc formed a small inhibition zone when cultured with *Streptococcus mutans and Staphylococcus aureus*. The inhibition zone of *Streptococcus mutans* was 10 ± 0.70 mm, while it was 1 ± 0.50 mm for *Staphylococcus aureus*. However, the DDMH disc showed significantly larger inhibition zones after culture with the same types of bacteria. The inhibition zone around the DDMH disc was 25 ± 0.70 mm with *Streptococcus mutans*, while it was 15 ± 0.44 mm with *Staphylococcus aureus*. This denoted a more significant antimicrobial effect of the DDMH disc versus the MTA.

**Table 3:** Kruskal–Wallis and Mann–Whitney statistical analysis for *streptococcus mutans* and *staph. aureus* inhibition zone of MTA versus DDMH

	Mean ± SD				*P* value
	IPM	Vancomycin	DDMH	MTA	
*Streptococcus mutans*	35 ± 0.44 mm	8 ± 1.78 mm P1 = 0.007	25 ± 0.70 mm P2 = 0.06 P4 = 0.008	10 ± 0.70 mm P3 = 0.006 P5 = 0.914 P6 = 0.007	*P* = 0.001***
*Staphylococcus aureus*	30 ± 0.89 mm	18 ± 0.44 mm P1 = 0.006	15 ± 0.44 mm P2 = 0.006 P4 = 0.08	1 ± 0.50 mm P3 = 0.007 P5 = 0.006 P6 = 0.006	*P* < 0.001***

Kruskal–Wallis, Mann–Whitney test. P^1^: comparison between IPM vs. vancomycin; P^2^: comparison between IPM vs. DDMH; P^3^: comparison between IPM vs. MTA; P^4^: comparison between vancomycin vs. DDMH; P^5^: comparison between vancomycin vs. MTA; P^6^: comparison between DDMH vs. MTA

Significance levels were set at *P < 0.05, **P < 0.01, ***P < 0.001

## Discussion

During reparative dentinogenesis, the odontoblasts and pulpal cells at the exposure site undergo necrosis and are replaced by new differentiated odontoblast-like cells [37]. In the present study, the biocompatibility and antimicrobial activities of DDMH were examined in comparison to MTA and the subsequent impact of the tested pulp capping materials on COL-I expression in DPSC as a first step in reparative dentin bridge formation.

This work focused on the comparison of DDMH versus the MTA that has been widely utilised for sealing the exposed pulpal tissue and the root canal system from surrounding tissues in several applications [18]. Since pulp capping materials will be in close contact with dental pulp, assessment of the effect of DDMH and MTA on DPSC was of utmost importance and is more translatable than using other cell types, which may not be native to the pulp area.

A complex of growth factors, including BMP-2, VEGF and COL-I are found in the dentin organic matrix and play an irreplaceable role in dentin formation and the regeneration of dentin-pulp-like tissue [38–42]. Nonetheless, DDM is problematic to be customised in DPC; therefore, DDM was fabricated into a hydrogel (DDMH) formed of DDM particles and sodium alginate to maintain its bioactivity.

It has been shown that the demineralisation reagent and the time of demineralisation of the dentin influence the dentin components. In this study, BMP-2, COL-I and VEGF were detected in demineralised dentin particles after different times of demineralisation, being significantly higher compared to control un-demineralised dentin particles. ELISA analysis of dentin particles after 3 days of demineralisation, which is considered complete demineralisation, revealed the presence of BMP-2, COL-I and VEGF in higher levels than all other treated or control dentin particles. A previous study demonstrated that dentin particles can withstand 7 days of demineralisation while preserving the structural integrity, but after 13 days of demineralisation, the dentin matrix collapses due to the destruction of the collagenous content [43]. In the current study, SEM analysis of dentin particles after demineralisation demonstrated that the longer the EDTA was left to react, the more exposure of dentinal tubules on the same surface area of the particles, dentinal tubules get enlarged, and the collagen matrix loosens, which can act as channels that release proteins essential for cell growth and differentiation [25, 44].

Different concentrations of tested eluants were studied to mimic the actual condition of pulp-capping agents affecting the pulp tissue. As shown in our study, the proliferative effect of all MTA eluants increased by decreasing their concentration, showing a dose-dependent cytotoxicity [45, 46]. On the other hand, different dilutions of DDMH eluants had no cytotoxic effect on DPSC; it is noteworthy that the viable cell number was higher in neat concentrations of DDMH eluants in comparison to control, concluding that increasing the concentration of DDMH eluants, an increase in cell proliferation was noted.

In this study, DPSC cultured with neat DDMH eluants had a higher mRNA expression for the COL-I gene than those cultured with neat MTA eluants. There was a gradual increase of COL-I gene expression in DPSC by increasing the concentration of DDMH eluants, indicating that DDMH eluants probably led to increased activity of pulp cells and differentiated to form odontoblast-like cells that increased the expression of COL-I. Similarly, COL-I gene expression was gradually increased in DPSC cultured in different concentrations of MTA eluants; however, the COL-I gene showed significant overexpression in DPSC cultured in DDMH eluants more than in MTA eluants at different concentrations. Concluding that neat concentrations of MTA eluants caused a cytotoxic effect on DPSC but remained capable of increasing the expression of the COL-I gene; however, neat concentrations of DDMH eluants promoted DPSC proliferation and upregulated COL-I gene expression [47]. This could explain why the bioactive molecules slowly released from the dentin matrix after demineralisation into the surrounding medium (eluants), and these proteins were used by DPSC for survival, proliferation, and differentiation.

Cell adhesion and proliferation are important factors for biocompatibility and are important for cell differentiation and tissue regeneration [48]. In our study, the AO staining revealed that the number of attached DPSC was markedly reduced after 7 days of incubation on the surface of MTA in comparison with DDMH. This finding may possibly be explained by the inhibited cell growth following contact with the MTA surface through the Ca^+2^ release from the material, along with the increase in pH. This agreed with other studies showing that MTA decreased the number of viable cells and induced apoptosis [49–52].

*Streptococcus mutans* and *Staphylococcus aureus* commonly significantly affect the initial and secondary pulpal lesions [53]. For this reason, our study examined the antibacterial effect of both pathogens against DDMH and MTA. This study detected DDMH’s strong antimicrobial activity against the tested organisms. The inhibitory effect of DDMH on *Streptococcus mutans* and *Staphylococcus aureus* was significantly better than MTA (*P* < 0.05). This agreed with another report in which the MTA demonstrated a small inhibition zone around the organisms, suggesting that the minimal antibacterial properties of MTA might be because of its alkalinity, which resulted in a reversible effect on bacterial cell membranes [54]. A study by Bhavana reported that the inhibition zones of *Streptococcus mutans* around MTA were large, which was opposite to our results [55]. Of note, the discrepancy of results between various reports could be because of the available nutrients, incubation periods, oxygen pressure levels, test methods, and laboratory tests utilised, as the agar plates may change the hydration degree of the tested MTA material.

Bioceramic materials are recommended as an alternative to MTA, Because of their superior physical, chemical, and biological qualities such as their demonstrated ability to promote cell differentiation, have osteoconductive effects, and lessen inflammation [56, 57]. Here, we demonstrated that DDMH, in addition to its antimicrobial action, enhanced DPSC viability and promoted cell adhesion, growth, and odontogenic differentiation by upregulating COL-I gene expression, making it a good substitute for pulp capping material. Nevertheless, our study had limitations. The agar diffusion method depends on the solubility and diffusion properties of the substances under evaluation, so materials with low solubility may have produced less satisfactory results. The odontogenic potential of DDMH eluants was evaluated in the current work using COL-I gene expression; however, more odontogenic markers need to be investigated. Furthermore, this study assessed the DDMH in contrast to a single capping material, MTA; we encourage other researchers to conduct additional investigations using different capping materials and evaluate the antimicrobial effectiveness of more capping agents than DDMH.

The ultimate goal for the tested material is to be suitable for clinical practice, and since our research was done in vitro, it was not possible to replicate the physiological circumstances of the oral cavity precisely. So, it is mandatory that a series of in vitro and in vivo experiments be conducted for a better understanding of the nature of the material and its capabilities to be effective in clinical use.

## Conclusion

This study evaluated the microstructure, biocompatibility and antimicrobial effect of DDMH. DDMH presented superior biocompatibility and antimicrobial properties compared to MTA and might be used as a promising pulp capping material.

## Data Availability

The data of our research are available with the corresponding author and can be provided whenever needed.
